# Effect of an 8-Week Yoga-Based Lifestyle Intervention on Psycho-Neuro-Immune Axis, Disease Activity, and Perceived Quality of Life in Rheumatoid Arthritis Patients: A Randomized Controlled Trial

**DOI:** 10.3389/fpsyg.2020.02259

**Published:** 2020-09-02

**Authors:** Surabhi Gautam, Manoj Kumar, Uma Kumar, Rima Dada

**Affiliations:** ^1^Lab for Molecular Reproduction and Genetics, Department of Anatomy, All India Institute of Medical Sciences, New Delhi, India; ^2^Department of Rheumatology, All India Institute of Medical Sciences, New Delhi, India

**Keywords:** rheumatoid arthritis, quality of life, inflammation, disease activity score, yoga-based lifestyle intervention, psycho-neuro-immune axis

## Abstract

Various external stressors and environmental challenges lead to the provocation of the immune system in autoimmune diseases like Rheumatoid arthritis (RA). The inappropriate immune response further triggers the cascade of inflammatory changes resulting in precipitation of symptoms and hampers quality of life (QOL). The underlying psycho-somatic component of the disease requires a holistic approach to its treatment dimension rather than the use of pharmacotherapy. The applicability of mind-body interventions has become essential in today’s fast-paced life. Yoga, a mind-body technique, alters the mind’s capacity to facilitate systemic functioning at multiple organ system levels. Hence, we conducted this study to evaluate the impact of 8 weeks of a yoga-based lifestyle intervention (YBLI) on psycho-neuro-immune markers, gene expression patterns, and QOL in RA patients on routine medical therapy. A total of 66 patients were randomized into two groups: yoga group or non-yoga group and were assessed for a panel of inflammatory cytokines (IL-6, IL-17A, TNF-α, and TGF-β), mind-body communicative markers (BDNF, DHEAS, β-endorphin, and sirtuin) and transcript levels of various genes (*IL-6, TNF-α, NFKB1, TGF*-β, and *CTLA4*). We assessed disease activity and QOL using the DAS28-ESR and WHOQOL-BREF questionnaire, respectively. Yoga group observed significant improvements in the levels of markers, which influenced the psycho-neuro-immune axis (*p* < 0.001) with an estimated effect size from small to medium range. In the yoga group, there was a significant reduction in DAS28-ESR (*p* < 0.001) and improvement seen in the physical health, psychological, social relationships domains (*p* < 0.001) of QOL, except environmental (*p* > 0.05). The yoga group showed downregulation of *IL-6, TNF*-α, and *CTLA4* and upregulation of *TGF*-β. These results suggest that a decrease in disease activity after yoga practice is associated with a significant reduction in inflammatory cytokines, the elevation of mind-body communicative markers, and normalization of various transcript levels, which improved QOL. Thus the adoption of YBLI improves clinical outcome in RA, and decreases systemic inflammation by its beneficial effects on psycho-neuro-immune axis and normalization of dysregulated transcripts. Thus YBLI may be used for RA patients as an adjunctive therapy.

## Introduction

Rheumatoid arthritis (RA) is a progressive autoimmune inflammatory disease characterized by arthritis of synovial-lined joints with variable systemic manifestation (Guo et al., 2018). It is the most commonly diagnosed systemic inflammatory arthritis, which severely impacts the quality of life (QOL) (Malm et al., 2017). Previous studies have shown an association of psychological stress with the onset and propagation of RA spanning both physical and mental domains of well-being (Margaretten et al., 2011; Matcham et al., 2013). Various articular and systemic manifestations in RA are associated with disability and reduced life expectancy (Cimmino et al., 2000). Multiple factors like loss of work productivity increased work disability, regular health care visits, long term treatment with disease-modifying anti-rheumatic drugs (DMARDs), and the need for joint replacement surgeries are associated with RA. They pose a significant burden on the individual, caregivers, and health care system (van Vilsteren et al., 2015).

Rheumatoid arthritis is a complex multifactorial disease, and includes a range of immune, neuroendocrine, and psychosocial variables (Glocker et al., 2006). The gene-environment interaction triggers the formation of autoantibodies like rheumatoid factor (RF), anti-citrullinated protein antibodies (ACPA), which leads to the onset and maturation of the disease (Malmström et al., 2017). Air pollutants such as silica, vehicular smoke, tobacco smoking, etc., lead to an activation of toll-like receptors located in the lung mucosal lining (Bauer et al., 2012; Malmström et al., 2017). Further, a calcium-mediated enzyme peptidyl arginine deiminase (PAD) triggers the process of citrullination. The binding of ACPAs to surface-expressed citrullinated proteins like Grp78 enhances the activity of nuclear factor kappa B (NFkB) and production of interleukin (IL)-6, and tumor necrosis factor-alpha (TNF-α) (Guo et al., 2018). NFkB is a protein complex that controls transcription of DNA, cytokine production and cell survival. IL-6 acts as pro-inflammatory cytokine and acts as mediator of acute phase response. TNF-α is a cell signaling cytokine involved in systemic inflammation.

The role of pleiotropic cytokine transforming growth factor-beta (TGF-β) is not well established, but the majority of studies found it to be anti-inflammatory and immune-modulatory (Sanjabi et al., 2009). There is an infiltration of leukocytes and pro-inflammatory mediators like IL-17A in the synovial compartment of the affected joint (Firestein and McInnes, 2017). The fulminant disease results in the formation of hyperplastic synovium, damaged cartilage, bony erosions, and extensive systemic inflammation. The essential neuro-endocrine pathway, the hypothalamic-pituitary-adrenal (HPA) axis, modulates the inflammatory response. There is a dysregulation of the HPA axis in RA patients (Cutolo et al., 2002). RA patients show adrenal insufficiency due to which they are unable to mount an appropriately enhanced glucocorticoid response to increased secretion of pro-inflammatory cytokines such as IL-1, IL-6, and TNF-α (Jessop and Harbuz, 2005). There is an insufficient vagal nerve activity in RA, which is the main nerve of the parasympathetic division of the autonomic nervous system and regulates metabolic homeostasis (Koopman et al., 2016, 2017). The vagus nerve stimulation targeting the inflammatory reflex modulates TNF production and reduces inflammation, hence inhibits cytokine production and attenuates disease severity in RA (Koopman et al., 2016). Various genetic, epigenetic, and environmental factors through distinct pathways and neurotransmitters are highly involved in altering the psycho-neuro-immune axis, resulting in the emergence of disease (González-Díaz et al., 2017). Significant stresses from different types of stimuli lead to pro-inflammatory load in RA patients due to defective stress response systems. There is bidirectional interaction between the nervous system and the immune system through soluble cytokines and mediators. The cytokines can traverse the nervous system either by crossing the blood-brain barrier, by diffusion through circumventricular organs or by directly stimulating the peripheral nerves (Otmishi et al., 2008). Stress triggers an aberrant immune response by the release of neuroendocrine hormones leading to precipitation of RA symptoms (Hassett and Clauw, 2010).

Rheumatoid arthritis patients need lifelong medicines to regulate the hyperactive immune system and live under constant anxiety and fear of fatal consequences like irreversible disability. So there is a dire need for its redressal by searching a cost-effective complementary form of treatment with minimal side-effects. Even the type of stressor, the individual’s response, and reactivity to stress may prove to be an important prognostic factor in disease onset and progression. Hence, the stress management therapies, along with the traditional pharmacotherapy interventions, may provide a greater sense of control over stress-induced RA symptoms and are capable of producing critical clinical benefits (Tolahunase et al., 2017, 2018; Gautam et al., 2019). Yoga is one such safe and effective therapy to improve physical functions along with stress reduction (Li and Goldsmith, 2012). Various studies have demonstrated the decisive role of yoga and meditation on stress, anxiety, and depression by eliciting a relaxation response that induces temporal transcriptome changes in energy metabolism, insulin secretion and inflammatory pathways (Li and Goldsmith, 2012; Bhasin et al., 2013; Pascoe et al., 2017). Yoga also improves joint flexibility, range of motion, posture, muscle strength, coordination, and thus self-competence and emotional resilience (Polsgrove et al., 2016). Various scientific reports summarized the beneficial effects of yoga on chronic disorders like hypertension, major depressive disorder, diabetes mellitus, aging, insomnia, glaucoma, obesity, and asthma. Yoga aids in the amelioration of mental stress and depression by improving the feeling of perceived well-being (Hagins et al., 2013; Rima et al., 2016; AlKhathami et al., 2017; Tolahunase et al., 2017, 2018; Dada et al., 2018; Sankalp et al., 2018). Yoga optimizes the levels of oxidative stress as it reduces ROS (reactive oxygen species) levels, reduces 8-OHdG (8-hydroxy-2′-deoxyguanosine) levels, and improves TAC (total antioxidant capacity) levels (Rima et al., 2016; Tolahunase et al., 2017, 2018; Gautam et al., 2019). Yoga also tends to normalize the levels of cortisol, improves brain-derived neurotrophic factor (BDNF), lipid profiles, diabetic profile, and inflammatory processes (Tolahunase et al., 2017, 2018; Raveendran et al., 2018). The pathophysiological association between depression and RA poses a similarity of alteration in biomarkers of both stress response and RA (Margaretten et al., 2011; Matcham et al., 2013).

The role of yoga as an effective intervention has been documented in the literature to assist in the management of chronic diseases like RA concerning its clinical symptoms like pain perception, stress management, disability outcomes, sleep quality, functional ability, QOL and psychosocial outcomes (Evans et al., 2011, 2013; Telles and Singh, 2012; Middleton et al., 2013; Gautam et al., 2019). Further exploration of a possible mode of action underlying the therapeutic effect of yoga at a cellular level is required and to establish how regular yoga practice affects the systemic biomarkers at the level of the psycho-neuro-immune axis to form a mind-body communication especially, in RA. We hypothesized that lowering of the stress response and elicitation of relaxation response by 8 weeks of a yoga-based lifestyle intervention (YBLI) in active RA patients reduces disease activity by altering the psycho-neuro-immune axis and its associated molecular and genetic markers. Current evidence is limited to establish a link between the multi-faceted dimensions of yoga and how it acts on the dysfunctional immune system and lowers the stress response. With this context in mind, the primary aim of the present study was to investigate the effects of 8-week YBLI on disease activity, psycho-neuro-immune axis markers, gene expression patterns, and QOL. We tested the biomarkers associated with the psycho-neuro-immune axis, including (a) inflammatory markers- IL-6, IL-17A, TNF-α, and TGF-β; (b) mind-body communicative markers- BDNF, DHEAS (dehydroepiandrosterone sulfate), β-endorphin and sirtuins. The expression patterns of the following genes investigated were: *IL-6, TNF-α, NFKB1* (nuclear factor kappa B subunit 1), *TGF*-β, and *CTLA4* (cytotoxic T-lymphocyte-associated protein 4). The inflammatory markers and inflammatory gene expression profile determine the level of systemic as well as local inflammation. The mind-body communicative markers are directly associated with the regulation of neuroplasticity (Tolahunase et al., 2018). In RA, the neuro-immunological mechanisms of immune dysfunction converge in the central nervous system (CNS) to alter molecular programs, neurogenesis, and plasticity and may lead to the development of co-morbid depression. As the immune system is an essential component of the physiological stress-sensing pathways and closely interacts with the body’s primary integrative mind-body communicative systems, including the HPA axis, in mutually regulatory feed-forward and feedback loops, so we included these markers.

## Materials and Methods

### Study Design

**FIGURE 1:**
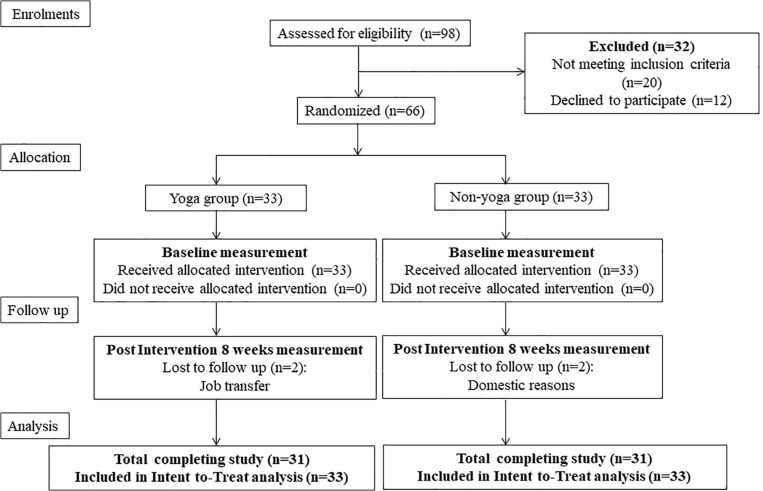
A consort flow diagram of the study.

The design of the present study was a prospective, single-blinded, randomized, controlled trial with active RA patients aimed at investigating the effects of 8 weeks of YBLI on disease activity, psycho-neuro-immune axis markers, gene expression patterns, and QOL. The study protocol complies with the Consolidated Standards of Reporting Trials (CONSORT) guidelines (Figure 1). The study initiated after obtaining ethical clearance (IECPG-211/24.02.2016) from the Institutional Ethics Committee of AIIMS, New Delhi, India, and registration under the clinical trials registry, India (REF/2016/01/010500). All the participants gave written informed consent before the commencement of the study protocol.

### Participants and Eligibility Criteria

Rheumatoid arthritis patients from the outpatient unit of the Rheumatology department of AIIMS, New Delhi, India, were screened for the following inclusion/exclusion criteria.

#### Inclusion Criteria for RA Patients

1. Patients of 18–60 years old diagnosed with RA as per 2010 ACR/EULAR RA classification criteria, whose disease activity score 28 erythrocyte sedimentation rate (DAS28-ESR) was >2.6 and were on routine medical treatment for at least 6 months.
2. No participation in a clinical trial within 4 weeks before the study.

#### Exclusion Criteria for RA Patients

1. Patients with any other autoimmune diseases other than RA or chronic systemic disease that could affect QOL.
2. Any history of administration of oral or intra-articular steroids in previous 6 months, or intake of any form of homeopathic, ayurvedic or herbal mineral supplementation.
3. Those who were already practicing yoga or meditation in any way.
4. Pregnant women and lactating mothers.
5. Patients who were physically unfit for yoga like who underwent any kind of joint replacement surgery, trauma involving the musculoskeletal system like a fracture, etc.
6. Those who were not willing to participate in the study.

### Sample Size Calculation

The sample size calculations for the study assumed to detect a standardized effect size [difference in mean change in DAS28-ESR between the two groups/pooled standard deviation (SD)] of 0.8 with a 95% confidence level and 80% power, considering the mean and SD of a previous study by Evans et al. (2011). The required sample size was 25 in both groups. Considering some loss to follow-up, we enrolled a total of 66 patients in the study and randomized into two groups- yoga group (33 patients) and non-yoga group (33 patients).

### Randomization

The sealed opaque envelopes consisted of the computer-generated random numbers, with the assistance of the web tool research randomizer^1^, used to randomize the patients into the yoga group or non-yoga group. The participants were not blinded to the study, whereas all investigators who interviewed patients, conducted experiments, and performed statistical analyses blinded to the group status of the patients.

### Intervention

A total of 66 participants randomized into two groups - yoga group (*n* = 33) and non-yoga group (*n* = 33). The study participants were all asked to undergo a clinical evaluation and provide a blood sample at baseline.

#### Yoga-Based Lifestyle Intervention (Yoga Group)

Participants of the yoga group were administered a standardized YBLI for 8 weeks, which was suitable for active RA patients in such a way that it did not cause any further irritation to already inflamed joints. The patients with deformed joints and limitations advised undergoing customized physical postures. The comprehensive YBLI program incorporated the components of Patanjali’s ashtanga yoga. Briefly, the intervention comprised of yogic practices including asanas (physical postures), pranayama (breathing techniques), dhyana (meditation), and savasana (relaxation techniques) followed by interactive counseling sessions on yoga, stress management, nutrition, as well as personal lifestyle management. YBLI was administered five times a week for 120 min duration per session for 8 weeks (Table 1). The sessions were conducted by the registered and well-qualified yoga instructors at Laboratory for Molecular Reproduction & Genetics, Department of Anatomy, AIIMS, New Delhi, India. As such, there was no home regimen to be followed for the yoga practice due to the extensive time commitment of each session, but the patients were not discouraged for the home practice. The patients were encouraged to incorporate yoga into their lifestyle after the end of the 8 weeks of intervention. The patients of this group continued with their routine DMARDs as per the prescription of rheumatologists.

**TABLE 1:** Details of activities in a single day session of the yoga-based lifestyle intervention (YBLI) program.

**S. No.**	**Practice to be done**			**Duration**
1.	Session preparation instructions			2 min
	Starting prayer			5 min
	Yogic sukshma vyama	Finger loosening, wrist loosening, elbow loosening, shoulder loosening, toe bending, ankle bending, knee cap tightening, patella movement, knee bending, and hip rotation		10 min
	Yogic sthool vyama	Rekhagati, Sarvaangpushti		
2.	Yogasana	Standing	Trikonasana	5 min
			Katichakrasana	
			Tadasana	
			Virabhadrasana	
		Sitting	Gomukhasana	5 min
			Paschim-utaanasana	
			Shashaankasana	
			Vakrasana	
		Prone	Ek-pada-shalabhasana	5 min
			Bhujangasana	
			Poorna- shalabhasana	
			Makarasana	
		Supine	Uttanapadasana	5 min
			Setubhandhasana	
			Pavanmuktasana	
			Matsyasana	
3.	Relaxation		Savasana	10 min
4.	Pranayama		Kapalbhati	20 min
			Ujjayi	
			Nadishodhana	
			Bhramari	
5.	Nada anusandhana, AUM- Aumkar recitation			3 min
6.	Dhyana (meditation)			15 min
7.	Shanti mantra- closing prayer			5 min
8.	Interactive session/self-directed learning			30 min
	Total			120 min

#### Usual Care Control (Non-yoga Group)

Patients assigned to the non-yoga group continued with their on-going usual medical care, which included DMARDs prescribed by the rheumatologists. The patients followed their normal day to day physical activities with no change in their daily routine for 8 weeks.

### Outcome Measures

#### Primary Outcome

The primary outcome was to measure the change in disease activity assessed by DAS28-ESR from baseline (day 0) to 8 weeks (Nishimoto and Takagi, 2010). The composite primary outcome was to evaluate the levels of psycho-neuro-immune axis markers including (a) inflammatory markers- (IL-6, IL-17A, TNF-α, and TGF-β); (b) mind-body communicative markers- BDNF, DHEAS, β-endorphin and sirtuins and the expression patterns of following genes: *IL-6, TNF-α, NFKB1, TGF*-β, and *CTLA4*.

#### Secondary Outcome

The secondary outcome was to measure the change in QOL of RA patients, which was measured by the validated generic WHO Quality of Life BREF (WHOQOL-BREF) questionnaire.

### Study Assessments

The assessment of study parameters was at day 0 (baseline) and 8th week (follow-up) of the intervention.

#### Measurement of Clinical Parameters

DAS28-ESR was used to assess the disease activity of RA patients. The DAS28-ESR consisted of four components: tender joint count, swollen joint count, visual analog scale (VAS) score of the patient’s global health, and ESR. In DAS28-ESR, a rating of ≤2.6 represents remission, >2.6 to 3.2 represents low disease activity, >3.2 to 5.1 represents moderate disease activity, and >5.1 represents high disease activity.

The assessment of QOL was made by the WHOQOL-BREF questionnaire which contains 26 original items, including 2-items examining an individual’s overall perception of the QOL and health; 24 items examining 4-domains (D1, physical; D2, psychological; D3, social; and D4, environmental) (Gholami et al., 2013). The physical domain has 7-items related to activities of daily living, dependence on medicinal substances and medicinal aids, energy, and fatigue, mobility, pain, and discomfort, sleep and rest and work capacity. The psychological domain has 6-items related to body image and appearance, negative feelings, positive feelings, self-esteem, spirituality, personal beliefs, thinking, memory, learning, and concentration. The social relationship domain has 3-items related to personal relationships, social support, and sexual activity. The environmental domain has 8-items on financial resources, freedom, physical safety, home environment, health and social care and their availability, opportunities for acquiring new information and skills, participation in recreation and leisure activities, physical environment, and transport. The WHOQOL-BREF questionnaire depicts a score ranging from 0 to 100, in which a higher score denotes a better QOL.

#### Measurement of Biochemical Marker Analysis

For the analysis of biochemical markers, 5 ml of the venous blood sample was collected under aseptic conditions by venipuncture and divided into three parts. From the first part (2 ml), the blood was allowed to clot, and the serum was separated. From the second part (2 ml), plasma was isolated by centrifugation at 2000 × *g* for 15 min at 4°C. RNA was isolated from the remaining third part (1 ml) of the blood. The serum and plasma were stored at −80°C until analyzed. ELISA kits used for the estimation of levels of IL-6 (Gen-Probe, Diaclone Diagnostic, France), IL-17A (Gen-Asia Biotech, China), TNF-α (Gen-Probe, Diaclone Diagnostic, France), BDNF (Raybiotech, Inc), DHEAS (Qayee Bio-Technology), β-endorphin (Phoenix Pharmaceuticals, Inc.) and sirtuin 1 (Qayee Bio-Technology). Serum TGF-β levels were estimated by a magnetic bead-based multiplex assay using Bio-Plex Pro TGF-β Assays (Bio-Rad Laboratories Inc., United States) according to manufacturer’s guidelines.

#### RNA Extraction, cDNA Synthesis, and qPCR

Total RNA was isolated from 1 ml of freshly obtained EDTA blood by TRIzol manual method. Complementary DNA (cDNA) synthesis was done by reverse transcribing 1,000 ng of RNA by using the iScript cDNA synthesis kit (Bio−Rad). The CFX96 real−time system (Bio−Rad, CA, United States) quantified the relative gene expression using Brilliant III Ultra−Fast SYBR Green qPCR Master Mix. The protocol for gene amplification was standardized at 35 cycles. Normalization of the amount of expressed mRNA used two internal housekeeping genes *36B4* and β-*actin.* The relative fold of gene expression was done by the 2^–ΔΔ*Ct*^ method. Each cDNA product formed was tested in triplicate. The primer sequences were as follows:

*IL-6*_Forward, 5′-GGCACTGGCAGAAAACAACC-3′;

*IL-6*_Reverse, 5′-GCAAGTCTCCTCATTGAATCC-3′;

*TNF-*α*_*Forward, 5′*-*ACCTCCGAGATGACACCATCA*-*3′;

*TNF-*α*_*Reverse, 5′-GGCACTCTGGCACATATTCAC*-3*′;

*NFKBI_*Forward, *5*′*-*CTGAGGCACTTCTGGGAGC*-*3′;

*NFKBI_*Reverse, 5′-CTCGAAAGTCTCGGAGCT-3′*;*

*TGF-*β*_*Forward, 5′-GAAGGGAGACAATCGCTTTAGC*-*3′;

*TGF-*β*_*Reverse, *5*′*-*TGTAGACTCCTTCCCGGTTGAG*-3*′;

*CTLA4*_Forward, 5′-GCTTTCTCCTCACAGCTGT-3′;

*CTLA4*_Reverse, 5′-TTTTCACATTCTGGCTCTGTT-3′;

*36B4*_Forward, 5′-AACATGCTCAACATCTCCCC-3′;

*36B4*_Reverse, 5′-CCGACTCCTCCGACTCTTC-3′;

β-*actin*_Forward, 5′-TGAGAGGGAAATCGTGCGTG-3′;

β-*actin*_Reverse, 5′-TGCTTGCTGATCCACATCTGC-3′.

### Statistical Analysis

All statistical analyses carried out on an intent-to-treat basis with the baseline observation carried forward approach using IBM SPSS Statistics for Macintosh, Version 25.0. (IBM Corp. Armonk, NY, United States) and GraphPad Prism (Version 6.01). Chi-square test and Fisher’s exact test compared the baseline characteristics between the two groups. The assessment of interaction effects among baseline parameters was carried out by mixed factorial design ANOVA (Peterson et al., 2017). For within-group analysis, paired *t*-test was used to study the difference between pre- to post-intervention for normally distributed data, or Wilcoxon signed-rank tests for continuous variables without normal distribution. For between-group analysis, the repeated measure ANOVA was used to study the intervention effects along with the interaction of time and group. The correlation matrix representing correlation coefficients between variables was calculated based on the Pearson correlation. In order to conduct the mediation analysis, the multiple regression analysis was used to determine the change in which variables significantly explained the associations with the dependent variable, i.e., change in pre and post intervention DAS28-ESR. A *p-*value < 0.05 was considered as statistically significant.

## Results

### Participants’ Baseline Characteristics

A total of 98 patients were screened for eligibility, out of which 66 were randomized into two groups (each group *n* = 33) (Figure 1). Baseline demographic characteristics, presenting symptoms, disease activity and QOL of all randomized participants are shown in Table 2. There were no significant differences in the baseline characteristics between the two intervention groups.

**TABLE 2:** Baseline characteristics.

**Variable**	**Group**		**χ^2^ value**	***t*-value**	***p*-value**
	**Yoga (*n* = 33)**	**Non-yoga (*n* = 33)**			
***Demographic characteristics***					
Age (years)	45.1 (8.7)	43.4 (9.3)	–	0.7484	0.4569
*Sex*					
Male	5 (15.2)	8 (24.2)	0.8621	–	0.5372
Female	28 (84.8)	25 (75.8)		–	
BMI (kg/m^2^)	25.9 (5.3)	24.6 (3.4)	–	1.126	0.2785
***Kuppuswamy’s Socioeconomic Status Scale***					
Upper	5 (16.0)	1 (3.2)	4.364	–	0.3590
Upper middle	10 (32.3)	12 (38.8)		–	
Lower middle	5 (9.7)	7 (22.5)		–	
Upper lower	10 (32.3)	12 (32.3)		–	
Lower	3 (9.7)	1 (3.2)		–	
***Presenting symptoms***					
Early morning stiffness (minutes)	22.4 (3.8)	23.5 (3.7)	–	0.1979	0.8437
Tender joint count (TJC)	6.0 (0.8)	6.4 (0.7)	–	0.4132	0.5143
Swollen joint count (SJC)	4.0 (0.7)	3.7 (0.5)	–	0.2668	0.6927
***Disease activity***					
DAS28-ESR	5.2 (1.1)	5.0 (0.8)	–	0.8603	0.3928
***Quality of life***					
WHOQOL-BREF Domain 1	43.3 (10.5)	44.8 (9.9)	–	0.6089	0.5447
WHOQOL-BREF Domain 2	47.2 (8.5)	48.0 (8.3)	–	0.4078	0.6848
WHOQOL-BREF Domain 3	49.0 (6.5)	49.2 (6.7)	–	0.1117	0.9114
WHOQOL-BREF Domain 4	53.5 (8.4)	53.7 (8.6)	–	0.0874	0.9306

*Data were described as frequency (%) for sex and mean (SD) for others; χ^2^, Chi-square value; *t*, *t*-test value.*

### Post-intervention Differences in Psycho-Neuro-Immune Axis Markers

As shown in Table 3, there was a significant change observed in the primary outcome, i.e., DAS28-ESR after the 8 weeks of the trial. There was a significant decrease found in DAS28-ESR (−0.8 ± 0.5; *p* < 0.0001 vs. −0.1 ± 0.6; *p* = 0.303) in yoga group as compared to non-yoga group after 8 weeks of intervention. The between group analysis showed a mean difference in DAS28-ESR [0.4, 95% CI (0.3 to 0.6)] with a significant interaction of group and time ($\eta_{\text{p}}^{2}$ = 0.246; *p* ≤ 0.001). A significant decline was noted in the levels of pro-inflammatory cytokine IL-6 [0.5, 95% CI (0.2 to 0.8); $\eta_{\text{p}}^{2}$ = 0.137; *p* = 0.002], IL-17A [0.1, 95% CI (−5.7 to 6.1); $\eta_{\text{p}}^{2}$ = 0.388; *p* ≤ 0.001] and TNF-α [1.1, 95% CI (0.3 to 1.9); $\eta_{\text{p}}^{2}$ = 0.311; *p* ≤ 0.001] in yoga group after 8 weeks, whereas the levels of anti-inflammatory cytokine TGF-β have shown a significant elevation [−3.3, 95% CI (−6.7 to −0.2); $\eta_{\text{p}}^{2}$ = 0.166; *p* = 0.001] after 8 weeks of YBLI. There was an upregulation in mind-body communicative markers with a significant mean difference with group and time interaction in BDNF [−2.0, 95% CI (−2.9 to −1.1); $\eta_{\text{p}}^{2}$ = 0.202; *p* < 0.001], DHEAS [−12.4, 95% CI (−14.5 to −10.3); $\eta_{\text{p}}^{2}$ = 0.602; *p* < 0.001], β-endorphin [−0.4, 95% CI (−0.7 to −0.8); $\eta_{\text{p}}^{2}$ = 0.431; *p* < 0.001] and sirtuins [−1.9, 95% CI (−2.9 to −0.9); $\eta_{\text{p}}^{2}$ = 0.362; *p* < 0.001] after 8 weeks of YBLI. Also, the change in mean within non-yoga group showed significantly increased levels of IL-17A (18.7 ± 23; *p* < 0.0001) and reduced levels of sirtuin 1 (−1.1 ± 2.2; *p* < 0.0001) and β endorphins (−0.7 ± 0.7; *p* < 0.0001) compared to baseline levels.

**TABLE 3:** Intent-to-treat analysis: means (SD) and results of within-group and between-group analysis of study outcomes (*n* = 66; yoga, 33; non-yoga, 33).

**Outcome**	**Yoga group**			**Non-yoga group**			**Between groups**	**Variance analysis/effects**			
	
	**Pre**	**Post**	**Δ Mean within-group**	**Pre**	**Post**	**Δ Mean within-group**	**Mean difference (95% CI)^b^**	**Time**		**Group × time**	
	
								***p*^b^-value**	**$\eta_{\text{p}}^{2}$**	***p*^b^-value**	**$\eta_{\text{p}}^{2}$**
***Disease activity score***											
DAS28-ESR	5.2 ± 1.1	4.5 ± 0.9	−0.8 ± 0.5	5.02 ± 0.8	4.9 ± 0.8	−0.1 ± 0.6	0.4 (0.3–0.6)	<0.001	0.372	<0.001	0.246
***Inflammatory markers***											
IL-6 (pg/ml)	3.5 ± 1.3	2.4 ± 1.7	−1.0 ± 1.1	3.8 ± 1.1	3.7 ± 1.5	−0.1 ± 1.4	0.5 (0.2–0.8)	0.001	0.153	0.002	0.137
IL-17A (pg/ml)	195.0 ± 31.0	176.6 ± 30.7	−19.0 ± 25.1	188.4 ± 38.6	207.2 ± 42.2	18.7 ± 23	0.1 (-5.7 – 6.1)	0.962	<0.001	<0.001	0.388
TNF-α (pg/ml)	17.0 ± 3.5	13.7 ± 3.8	−3.3 ± 3.0	17.5 ± 6.9	18.5 ± 5.4	1.0 ± 3.4	1.1 (0.3–1.9)	0.006	0.113	<0.001	0.311
TGF-β (ng/ml)	41.9 ± 16.8	51.3 ± 15.5	93.9 ± 13.6	44.4 ± 14.4	41.8 ± 15.1	−26.2 ± 13.7	−3.3 (-6.7 – 0.2)	0.049	0.059	0.001	0.166
***Mind-body communicative markers***											
BDNF (ng/ml)	13.3 ± 3.9	17.2 ± 6.1	3.8 ± 4.5	13.5 ± 4.3	13.8 ± 4.7	0.2 ± 2.4	−2.0 (-2.9 – -1.1)	<0.001	0.247	<0.001	0.202
DHEAS (ng/ml)	37.4 ± 5.7	60.3 ± 8.6	22.8 ± 10.6	35.3 ± 4.1	37.3 ± 5.3	2.0 ± 5.9	−12.4 (-14.5 – -10.3)	<0.001	0.683	<0.001	0.602
β-endo (ng/ml)	3.7 ± 1.3	5.3 ± 1.7	1.5 ± 1.7	3.8 ± 0.6	3.1 ± 0.7	−0.7 ± 0.7	−0.4 (-0.7 – -0.8)	0.015	0.089	<0.001	0.431
Sirtuin 1 (ng/ml)	31.8 ± 1.8	36.8 ± 5.9	5.0 ± 5.3	30.7 ± 2.4	29.6 ± 2.4	−1.1 ± 2.2	−1.9 (-2.9 – -0.9)	<0.001	0.187	<0.001	0.362
***WHOQOL-BREF score***											
D1	43.3 ± 10.3	56.4 ± 10.5	13.1 ± 7.8	44.8 ± 9.9	43.3 ± 7.3	−1.4 ± 7.2	−5.8 (-7.6 – -3.9)	<0.001	0.384	<0.001	0.493
D2	47.2 ± 8.5	60 ± 5.4	12.8 ± 7.4	48.0 ± 8.3	45 ± 9.9	−3.0 ± 6.8	−4.9 (-6.6 – -3.1)	<0.001	0.329	<0.001	0.561
D3	49.0 ± 6.5	53.5 ± 6.7	4.5 ± 4.5	49.2 ± 6.6	46.7 ± 9.1	−2.5 ± 5.4	−1.0 (-2.2 – 0.2)	0.113	0.039	<0.001	0.333
D4	53.5 ± 8.3	54.3 ± 7.3	0.7 ± 3.8	53.7 ± 8.5	52.7 ± 7.6	−0.9 ± 3.8	0.12 (-0.8 – 1.1)	0.799	0.001	0.078	0.048

*Δ mean within-group, mean score change within the group; *p*^*b*^, *p*-value with Bonferroni correction; $\eta_{p}^{2}$, partial eta square value representing the effect size; WHOQOL-BREF Domains, D1, physical; D2, psychological; D3, social; and D4, environmental.*

### Post-intervention Differences in Quality of Life

**FIGURE 2:**
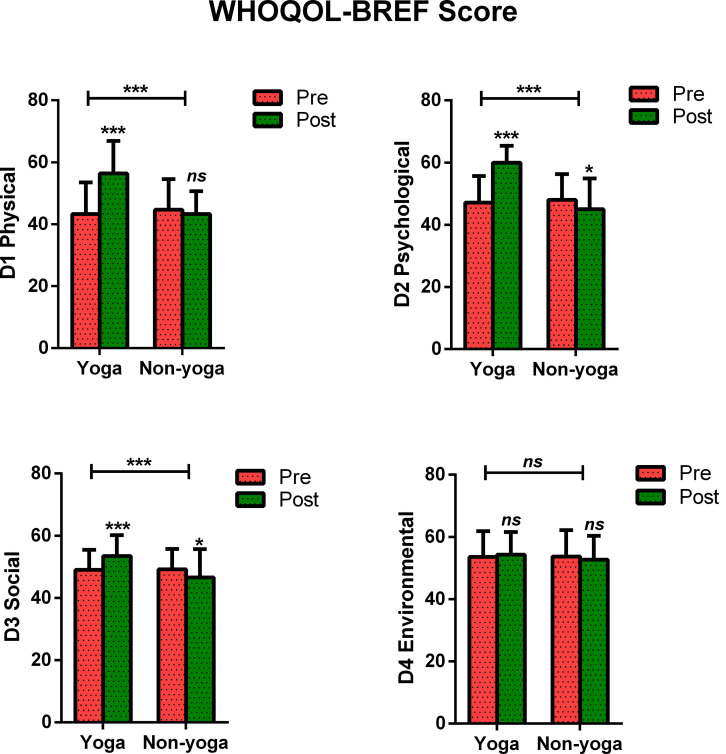
Between and within-group analysis of WHOQOL-BREF Scores in yoga group and non-yoga group. *p*-value (ns = *p* > 0.05; *p ≤ 0.05; **p ≤ 0.01; ***p ≤ 0.001).

The change in mean scores of QOL for physical domain D1 (13.12 ± 7.8 vs. −1.4 ± 7.2; *p* < 0.0001), psychological domain D2 (12.8 ± 7.4 vs. −3.0 ± 6.8; *p* < 0.0001), social domain D3 (4.5 ± 4.6 vs. −2.5 ± 5.4; *p* < 0.0001) were found to be statistically significant in yoga group over non-yoga group. A trend toward improvement was noted in environmental domain (domain D4) scores (0.72 ± 3.8 vs. −0.96 ± 3.8; *p* = 0.078) in yoga group as compared to the control group (Figure 2 and Table 3). The variance analysis showed a significant mean score difference in QOL for physical domain D1 [−5.8, 95% CI (−7.6 to −3.9); $\eta_{\text{p}}^{2}$ = 0.493; *p* < 0.001], psychological domain D2 [−4.9, 95% CI (−6.6 to −3.1); $\eta_{\text{p}}^{2}$ = 0.561; *p* < 0.001], social domain D3 [−1.0, 95% CI (−2.2 to 0.2); $\eta_{\text{p}}^{2}$ = 0.333; *p* < 0.001] in yoga group as compared to non-yoga group. The environmental domain D4 did not show any statistically significant mean score difference in both the groups [0.12, 95% CI (−0.8 −1.1); $\eta_{\text{p}}^{2}$ = 0.048; *p* = 0.078] (Table 3).

**FIGURE 3:**
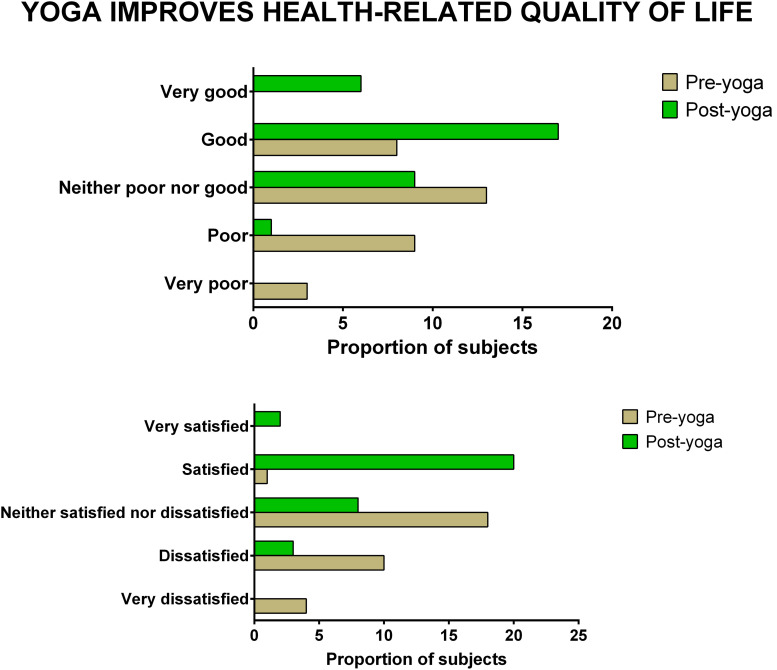
The improvement in overall quality of life and overall quality of health after yoga-based lifestyle intervention.

The data showed significant improvement in the overall QOL (item 1) from baseline to week 8, as assessed by the WHOQOL-BREF questionnaire. At the end of the study, no participant had a poor perception of overall QOL after the YBLI, and most of the participants perceived an overall good QOL. This shift in scores from baseline was statistically significant in the yoga group (*p* = 0.0007) (Table 4 and Figure 3). In-line, the results for the overall quality of health (item 2) showed that none of the participants was very dissatisfied after the YBLI, and most participants were satisfied. This shift in scores from baseline was statistically significant in the yoga group (*p* < 0.0001) (Table 5 and Figure 3).

**TABLE 4:** Pre–post comparison of World Health Organization quality of life-BREF responses: overall perception of quality of life (item 1) in RA patients.

**Variable**	**Pre-intervention (baseline)**	**Post-intervention (8th week)**					***p*-values**
		**Very poor**	**Poor**	**Neither poor nor good**	**Good**	**Very good**	
**Yoga group**							
Very poor	3	0	1	2	0	0	**0.0007**
Poor	9	0	0	5	3	1	
Neither poor nor good	13	0	0	2	9	2	
Good	8	0	0	0	5	3	
Very good	0	0	0	0	0	0	
**Non-yoga group**							
Very poor	3	0	3	0	0	0	**0.7084**
Poor	9	1	6	2	0	0	
Neither poor nor good	12	0	1	8	3	0	
Good	9	0	0	1	7	1	
Very good	0	0	0	0	0	0	

*Data expressed as the number of participants (*n*).*

**TABLE 5:** Pre–post comparison of World Health Organization quality of life-BREF responses: Overall perception of quality of health (item 2) in RA patients.

**Variable**	**Pre-intervention (baseline)**	**Post-intervention (8th week)**					***p*-values**
		**Very dissatisfied**	**Dissatisfied**	**Neither dissatisfied nor satisfied**	**Satisfied**	**Very satisfied**	
**Yoga group**							
Very dissatisfied	4	0	2	1	1	0	**<0.0001**
Dissatisfied	10	0	1	3	5	1	
Neither dissatisfied nor satisfied	18	0	0	4	14	0	
Satisfied	1	0	0	0	0	1	
Very satisfied	0	0	0	0	0	0	
**Non-yoga group**							
Very dissatisfied	2	1	0	1	0	0	**0.1775**
Dissatisfied	17	2	7	5	3	0	
Neither dissatisfied nor satisfied	11	0	2	6	3	0	
Satisfied	3	0	0	0	1	2	
Very satisfied	0	0	0	0	0	0	

*Data expressed as the number of participants (*n*).*

### Gene Expression Analysis

**FIGURE 4:**
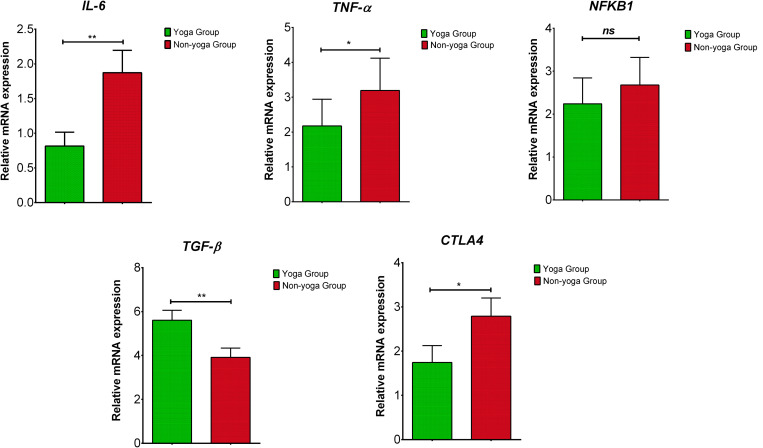
The relative m RNA expression levels of dysregulated transcripts in yoga group and non-yoga group. *IL-6* (interleukin-6), *TNF*-α (tumor necrosis factor alpha), *NFKB1* (nuclear factor kappa B subunit 1), *TGF*-β (transforming growth factor beta), and *CTLA4* (cytotoxic T-lymphocyte-associated protein 4); p value (ns = *p* > 0.05; *p ≤ 0.05; **p ≤ 0.01; ***p ≤ 0.001).

The results showed significant downregulation in relative mRNA expression levels of *IL-6* (*p* = 0.001), *TNF*-α (*p* = 0.0271) and *CTLA-4* (*p* = 0.0129) in the yoga group as compared to the non-yoga group (Figure 4). The mean axis fold change of these transcripts was as follows in the yoga vs. non-yoga group: *IL-6* (−2.6 vs. 0.4), *TNF*-α (−2.7 vs. 1.35), and *CTLA-4* (−0.5 vs. 2.2), respectively. The mRNA expression levels of *NFKB1* were not found to be different statistically (*p* = 0.9137) with a mean axis fold change of 0.6 vs. 1.1 in the yoga vs. non-yoga group (Figure 4). In the yoga group, the mRNA expression levels of *TGF*-β (*p* = 0.0052) were significantly upregulated with the mean axis fold change of 5.5 vs. 3.8 in the yoga vs. non-yoga group (Figure 4).

### Group × Gender Interactions

**FIGURE 5:**
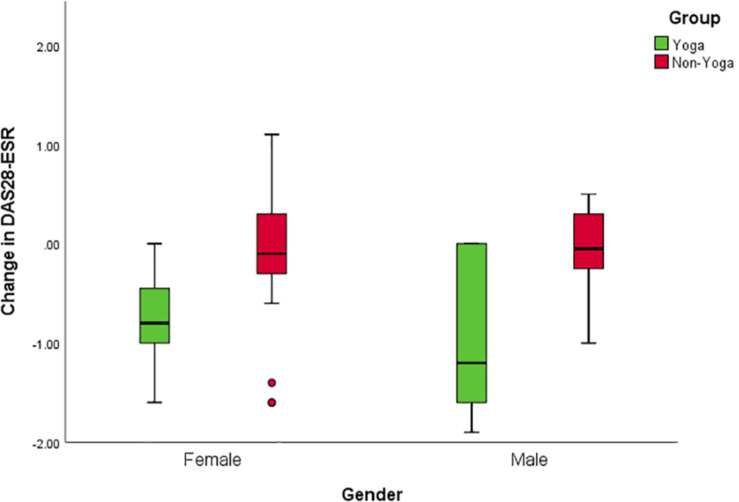
Gender interactions for change in disease activity after the intervention. Mean change of DAS28-ESR score with 95% CI for males in the yoga and the control groups; *p* = 0.0407 for between-group differences of change in the study, adjusted for baseline value. Mean change of DAS28-ESR score with 95% CI for females in the yoga and the control groups; *p* = 0.0003 for the between-group difference of change on the study, adjusted for baseline value.

There was no significant difference in mean DAS28-ESR values between men and women at baseline (DAS28-ESR values 5.1 ± 0.1 and 5.2 ± 0.3 mean ± SD, respectively). Separate analyses for males and females were performed to overcome baseline violations in disease activity and to explore further specific gender effects. Interaction effects including group and gender indicated differential responses to YBLI for women for DAS28-ESR [*F*(1,27) = 70.3; *p* < 0.001], BDNF [*F*(1,27) = 22.6; *p* < 0.001], sirtuins [*F*(1,27) = 24.3 *p* < 0.001], IL-17A [*F*(1,27) = 15.2; *p* = 0.001], IL-6 [*F*(1,27) = 23.2; *p* < 0.001], TNF-α [*F*(1,27) = 38.5; *p* < 0.001], TGF-β [*F*(1,27) = 10.3; *p* = 0.003]. Clinical improvement was more significant for the women in yoga group [mean between-group difference of change [95% confidence interval (CI)]: female = 0.6, 95% CI (0.3 to 0.9) *p* = 0.0003; male = 0.8, 95% CI (0.04 to 1.7) *p* = 0.0407 (Figure 5).

### Correlation Between Psycho-Neuro-Immune Markers, Gene Expression and Quality of Life

The analysis showed a correlation between the pre and post-intervention change among the tested variables (Table 6). The improvement of DAS28-ESR was correlated positively with the changes of WHOQOL-BREF scores and mind-body communicative markers (BDNF, DHEAS, β-endorphin, and sirtuin) but negatively with the changes of inflammatory markers (IL-6, IL-17A, TNF-α). However, both the groups showed non-significant relationships with the pre and post intervention change of different variables with respect to change in DAS28-ESR (Table 7). The fact that the observed *p*-values did not fall below the established alpha level of 0.05 which indicated that the association among the measured variables not significantly affected the dependent variable by the inclusion of the mediators in the model; in other words, there is no evidence of mediation.

**TABLE 6:** Correlations analysis of pre-post differences (changes) between primary and secondary outcomes (n = 66)..

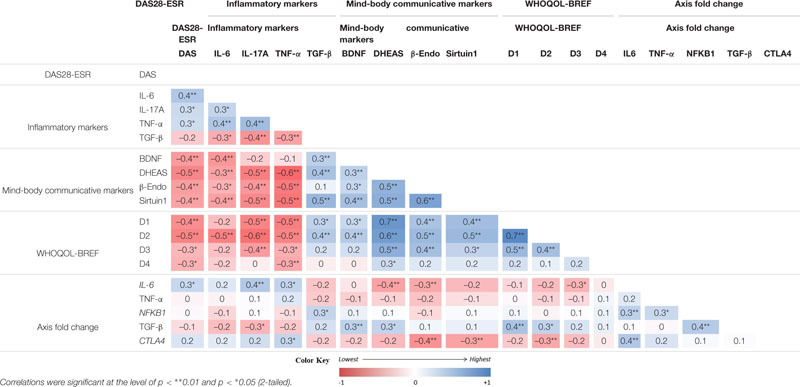

**TABLE 7:** Predictors of change in DAS28-ESR with group interactions.

**Group**	**Variable**	**Unstandardized co-efficient**		**Standardized co-efficient**	***t***	***p*-value**
		***B***	**Standard error**	**β**		
**Yoga**						
	Constant	–0.261	0.286		–0.911	0.373
***Inflammatory markers***						
	Δ IL-6	0.036	0.120	0.073	0.305	0.764
	Δ IL-17A	0.004	0.005	0.176	0.803	0.431
	Δ TNF-α	–0.071	0.040	–0.392	–1.769	0.092
	Δ TGF-β	1.476*E*−06	0.000	0.037	0.158	0.876
***Mind-body communicative markers***						
	Δ BDNF	–0.025	0.028	–0.209	–0.892	0.383
	Δ DHEAS	–0.013	0.011	–0.264	–1.277	0.216
	Δβ-*endo*	–0.063	0.066	–0.204	–0.956	0.350
	Δ Sirtuin 1	–0.014	0.026	–0.140	–0.546	0.591
***WHOQOL-BREF score***						
	Δ D1	–0.040	0.021	–0.572	–1.902	0.072
	Δ D2	0.034	0.022	0.464	1.576	0.131
	Δ D3	0.007	0.026	0.059	0.267	0.792
	Δ D4	–0.032	0.029	–0.232	–1.125	0.274
**Non-yoga**						
	Constant	–0.071	0.224		–0.317	0.754
***Inflammatory markers***						
	Δ IL-6	0.035	0.136	0.078	0.258	0.799
	Δ IL-17A	0.000	0.006	0.007	0.030	0.977
	Δ TNF-α	0.038	0.070	0.203	0.539	0.596
	Δ TGF-β	6.091*E*−06	0.000	0.132	0.446	0.661
***Mind-body communicative markers***						
	Δ BDNF	–0.091	0.085	–0.348	–1.070	0.297
	Δ DHEAS	–0.006	0.023	–0.059	–0.267	0.792
	Δβ-*endo*	0.082	0.196	0.098	0.420	0.679
	Δ Sirtuin 1	0.014	0.066	0.049	0.215	0.832
***WHOQOL-BREF score***						
	Δ D1	0.012	0.020	0.133	0.572	0.574
	Δ D2	–0.024	0.023	–0.259	–1.055	0.304
	Δ D3	0.017	0.025	0.143	0.658	0.518
	Δ D4	–0.032	0.036	–0.190	–0.872	0.393

*Δ indicates pre–post intervention change.*

## Discussion

In the present study, we found that 8 weeks of YBLI significantly reduced disease activity, normalized the biomarkers of the psycho-neuro-immune axis with associated changes in gene expression patterns, and improved QOL. With a decline in DAS28-ESR, we observed reduced levels of inflammatory markers, elevated mind-body communicative markers associated with the psycho-neuro-immune axis in the yoga group. The yoga group showed the downregulation of *IL-6, TNF*-α, and *CTLA4* and upregulation of *TGF*-β. Our study also highlights a significant improvement in three of four domains of QOL after 8 weeks of YBLI. There was an improvement in various molecular and genetic markers associated with the psycho-neuro-immune axis. Also, there was a favorable clinical outcome of RA with a reduction in disease activity and improvement in QOL. These findings suggest that yoga may induce molecular remission in RA by influencing its pathobiology by targeting mind-body communications.

In the present study, the impact of YBLI was beneficial as there was a significant reduction in pro-inflammatory markers (IL-6, IL-17A, and TNF-α) and elevation in anti-inflammatory markers (TGF-β). Anti-inflammatory effects of yoga try to re-establish optimal immune-homeostasis and bring about natural immunological tolerance to overcome the autoimmune conditions (Gautam et al., 2019). Our results are in concordance with other studies that demonstrated yoga’s potential role in reducing sympathetic over-activity, increasing vagal activity, and hence lowering inflammation (Rajbhoj et al., 2015; Vijayaraghava et al., 2015; Shete et al., 2017). Yoga and meditation have various physiological implications that help in reducing psychological stress parameters, which help in healthy aging and cellular longevity (Tolahunase et al., 2017, 2018). Yoga intervention leads to reduced levels of oxidative stress and increased telomerase activity in apparently healthy individuals (Tolahunase et al., 2017). The long term practice of meditation exhibited downregulation of T cell receptor signaling and inflammatory response pathways associated with *NFKB, RELA, TNFR2* transcription factors (Bhasin et al., 2013). *NFKB* is a potential bridge between inflammation, psychosocial stress, aging, oxidative cellular activation (Lawrence, 2009). Our results show a non-significant decline in relative mRNA expression of *NFKB1* in the yoga group as compared to the non-yoga group after a short-term intervention of 8 weeks. However, we observed a declining trend of *NFKB1* expression in the yoga group as compared to the non-yoga group.

IL-6 is a pleiotropic cytokine encoded by the *IL-6* gene located on chromosome 7, plays a crucial role in the pathogenesis of RA, and has a strong positive correlation with the disease activity and joint destruction. It mediates its pro-inflammatory actions by binding to its transmembrane or soluble receptors and further activating gp130, a transmembrane protein, to initiate the IL-6 signaling pathway. IL-6 signaling also activates various transcriptional factors via the JAK-STAT signaling pathway, which leads to proliferation, differentiation, and activation of CD4+ T cells (Su et al., 2017). IL-6 in the presence of TGF-β also promotes the differentiation of Th17 cells, which secrete IL-17A, another potent pro-inflammatory cytokine. At the same time, it inhibits TGF-β induced differentiation of regulatory T (T reg) cells (Martinez et al., 2008). Hence, IL-6 creates an imbalance of immune-homeostasis by increasing the Th17 population over T reg cell populations. IL-6 is also a potent activator of the HPA axis, induces acute phase reactions, and causes the production of autoantibodies by stimulating B cells (Venihaki et al., 2001). In RA, there is an autonomic dysfunction which is represented by a low parasympathetic activity. The vagus nerve plays an important role in regulating inflammation as it decreases production of pro-inflammatory cytokines such as IL-6, TNF-α, etc. and inhibits the migration of immune cells to sites of inflammation (Bonaz et al., 2017). Heart rate variability (HRV) is a reliable index of cardiac-vagal regulation (Breit et al., 2018). HRV is inversely related to the levels of inflammatory markers (Bonaz et al., 2017; Breit et al., 2018). The vagus nerve stimulates the HPA axis through its afferent fibers to release glucocorticoids by the adrenal glands and is also involved in the cholinergic anti-inflammatory pathway through a vago-vagal reflex. The sympathetic nervous system and the vagus nerve interact both through a vago-sympathetic pathway involving vagal afferent fibers and a vago-splenic pathway through vagal efferent fibers (Cooper et al., 2015). The modulation of vagus nerve is able to improve various inflammatory disorders, like irritable bowel syndrome, inflammatory bowel diseases, RA, obesity, and pain, etc. which is possible via pharmacological manipulation, vagus nerve stimulation, nutritional therapies, yoga intervention and physical exercise (Bonaz et al., 2017). In inflammatory conditions, the HPA axis is continuously under stress. Stressors/pro-inflammatory cytokines trigger the hypothalamus to release corticotrophin-releasing hormone (CRH). CRH, in turn, acts on anterior pituitary and induces the release of adrenocorticotropic hormone (ACTH), which in turn stimulates the adrenal gland to produce and release cortisol. Cortisol counteracts the stressor and reduces the existing inflammation (Silverman et al., 2005). But due to continuous inflammatory stimuli, the HPA axis is sensitized, and there is an initiation of negative feedback regulation of cortisol on the hypothalamus and anterior pituitary. This condition leads to adrenal insufficiency. Various studies have shown that yoga modulates stress and inflammation by suppressing hypersensitive HPA axis and modulating cortisol levels (Arora and Bhattacharjee, 2008).

Rheumatoid arthritis patients experience clinical symptoms like joint stiffness, pain, and functional disability mostly in the early morning as these symptoms closely follow the circadian rhythm of the pro-inflammatory cytokines (Sierakowski and Cutolo, 2011). Human pro-inflammatory cytokine production also exhibits diurnal rhythmicity. When the levels of pro-inflammatory cytokines are highest during the night, and early morning, there exists an imbalance in serum cortisol level (lowest) and serum melatonin level (highest). There is an increase in nocturnal anti-inflammatory cortisol secretion, which is insufficient to suppress the ongoing inflammation, resulting in the morning symptoms characteristic of RA. This imbalance of anti-inflammatory effects exerted by cortisol and pro-inflammatory effects by melatonin plays a crucial role in the pathogenesis of RA (Cutolo et al., 2003; Cutolo, 2019). A low dosage of corticosteroid replacement therapy aids in relieving aggravated clinical symptoms. Also, melatonin inhibitors may prove to be therapeutic for RA in relieving the symptoms associated with RA (Cutolo et al., 2003). The previous study from our laboratory demonstrated a significant decline in the cortisol levels after 12 weeks of YBLI in a healthy population as well as in depression patients (Tolahunase et al., 2017, 2018). The therapeutic role of yoga and the time of its administration during the day become relevant to study its impact on circadian rhythm in RA patients. Our study also demonstrates that yoga normalizes the circulating levels (IL-6, IL-17A, TNF-α) and mRNA transcript levels of pro-inflammatory cytokines (*IL-6, TNF*-α), hence reduces systemic and local inflammation in RA. Studies suggest that the vagus nerve has a profound anti-inflammatory effect and its activity is increased by yoga as indexed by HRV. Yoga also brings a balance between sympathetic and parasympathetic limbs in inflammatory conditions and restores autonomic reflex regulatory mechanisms.

TGF-β is another important pleiotropic cytokine which by its regulatory functions, helps to maintain immune-homeostasis and induces peripheral tolerance. TGF-β supports the survival of naturally occurring T reg cells and promotes the differentiation of induced T reg cells with IL-2 and retinoic acid. The protective nature of T reg cells keeps a track on the hyperactive immune system and prevent autoimmunity (Gonzalo-Gil and Galindo-Izquierdo, 2014). The results from our study showed that yoga holds the immune-regulatory potential and reduces the severity of RA by re-establishing immunological tolerance. CTLA4 is a co-inhibitory molecule that modulates the CD28: CD80/86 co-stimulation signal, thus inhibits T cell response. CTLA4 has a stronger affinity to bind with CD80 and CD86 and sends inhibitory signals to T cells. CTLA4 is also found in T regs and contributes to their inhibitory function. The blockade of CTLA-4 reduces the process of angiogenesis, suppresses *NFKB* activation, and promotes osteoclastogenesis (Cutolo et al., 2016). Our results have shown that the yoga group showed significant downregulation of mRNA expression levels of *CTLA4* as compared to the non-yoga group, further confirming the immune-regulatory role of YBLI.

We have also seen a marked improvement in the mind-body communicative markers, which is indicated by increased levels of BDNF, DHEAS, β endorphins, and sirtuin-1, followed by 8 weeks of YBLI in RA patients. YBLI elevates the levels of BDNF, a cardinal biomarker of neuroplasticity. BDNF possesses neurotrophic and neuroprotective abilities (Naveen et al., 2016; Gagrani et al., 2018). Our study confirms that YBLI increases BDNF and improves mind-body communications that regulate neuroplasticity and reduce associated stress cascade. DHEAS has neuroprotective, antioxidant, and anti-inflammatory properties. There is dysregulation of DHEAS levels in RA patients (Maninger et al., 2009). Previous studies have shown that DHEAS levels are upregulated in RA patients when administered with TNF antagonists and improved adrenal functioning (Schmidt et al., 2005; Maninger et al., 2009). There is a significant elevation in the DHEAS levels after 8 weeks of YBLI, which helps in the maintenance of emotional regulation, neurocircuits, and memory. Upregulated DHEAS levels after yoga intervention also reduces depression severity, as seen in primary depression patients (Tolahunase et al., 2018). β endorphins are endogenous morphine secreted by the pituitary and reached all the tissues via diffusion. This neuro-hormone acts as a neuro-regulator and circulating levels of which tend to increase with a particular form of physical activity of sufficient intensity and duration (Schwarz and Kindermann, 1992). Hence, yoga also possesses the potential to improve the levels of β endorphins and develops a positive feeling with a sense of happiness, well-being, and security in already depressed RA patients. Yoga also resulted in the upregulation of sirtuins, which promote cellular longevity by deactivation of p53 mediated pathways that are involved in apoptosis, growth arrest, or senescence in response to cellular stress (Tolahunase et al., 2018; Gautam et al., 2019). Our findings suggest that yoga has the ability to reorganize the structure and function of nervous system which was manifested at genetic and biochemical levels by downregulation of inflammatory markers, upregulation of markers of neuroplasticity and reduction in DAS28-ESR scores. In our study, it was found that clinical improvement was more significant for the women in yoga group in terms of DAS28-ESR and various other markers, i.e., BDNF, sirtuins, IL-17A, IL-6, TNF-α, and TGF-β. Women tended to show higher reductions in disease activity and depression severity. This may be due to transitory and fluctuating sex hormone levels that induce the continuous functional adaptation of the CNS throughout the lifespan, particularly in females (Juraska et al., 2013). It has also been observed that neurogenesis is higher in female than in males, probably because of the variations in gonadal hormones as estrogens modulate spatial and contextual memory in females (Duarte-Guterman et al., 2015). The results of this study also showed significant improvement in the overall QOL as well as health after the YBLI, which improvement in the physical, psychological, and social domain scores of WHOQOL-BREF questionnaire. Yoga improved the physical fitness, psychological health, and feeling of general well-being, among the patients practicing in the yoga.

The limitations of the study included lack of an active control group as the non-yoga group was not exposed to any equal attention control intervention and was only on drug therapy as compared to the active yoga intervention group. Hence, the inclusion of such a group would further rule out the therapeutic findings explicitly attributed to the yoga intervention. The number of males was less in both yoga and non-yoga group which was attributed to the higher prevalence of RA in females than males (3:1) (van Vollenhoven, 2009). The change in the levels of psycho-neuro-immune markers was more significant for the women in yoga group as compared to non-yoga group which can also be attributed to small sample size of males in both the groups. The sample size in our study was small; hence the correlation statistics might improve with the inclusion of greater numbers. The study design was single-blinded, which had limitations of an increased likelihood of bias, participant dissatisfaction with non-treatment status, and preconceived notions about treatment. Each session of YBLI lasted for 2 hour every day under the supervision of a certified yoga instructor, which is difficult to sustain regularly for a long term RA management or home practice regimen. For long term sustainable improvements of this chronic debilitating disease, there is need for daily yoga practice and a follow-up period. Future research should explore factors contributing to discontinued practice and whether yoga at home is a suitable substitute for the completion of a yoga program. Further we also plan to elucidate the importance of anti-inflammatory effect of the vagus nerve by measuring the HRV in RA patients after YBLI. In our study, there was no follow-up period, so it was difficult to predict how quickly participants returned to baseline levels of all biomarkers. In future studies, we plan to include a substantial sample size with long term follow-ups to study the long term benefits of the yoga practice.

In conclusion, yoga possesses an immune-modulatory potential which regulates the psycho-neuro-immune axis, reduces disease activity, and improves QOL in RA patients. YBLI maintains immunological tolerance by modulation of inflammatory processes. YBLI also normalizes the dysregulated transcripts associated with the stress pathway. YBLI may be beneficial for RA patients as adjunctive therapy, which enhances physical functioning and improves psychological health.

## Data Availability Statement

The datasets generated for this study are available on request to the corresponding author.

## Ethics Statement

The studies involving human participants were reviewed and approved by Institute Ethics Committee, AIIMS, New Delhi, India. The patients/participants provided their written informed consent to participate in this study.

## Author Contributions

SG designed the study, performed the experiments, analyzed the data, and wrote the manuscript. MK helped SG in standardizing amplification protocols. UK provided the patient samples and clinical data. RD conceived, conceptualized the study, thoroughly reviewed, edited, and finalized the manuscript. All authors contributed to the article and approved the submitted version.

## Conflict of Interest

The authors declare that the research was conducted in the absence of any commercial or financial relationships that could be construed as a potential conflict of interest.

## Footnotes

1 https://www.randomizer.org/
